# Supplementary material for: Induction Therapies Determine the Distribution of Perforin and Granzyme B Transcripts in Kidney Transplant Recipients
Source: Biomedicines. 2024 Jun 5;12(6):1258. doi: 10.3390/biomedicines12061258 (PMC11200803; doi:10.3390/biomedicines12061258)
Supplement: Supplementary file 1 [file biomedicines-12-01258-s001.zip › biomedicines-3022719-supplementary.pdf]

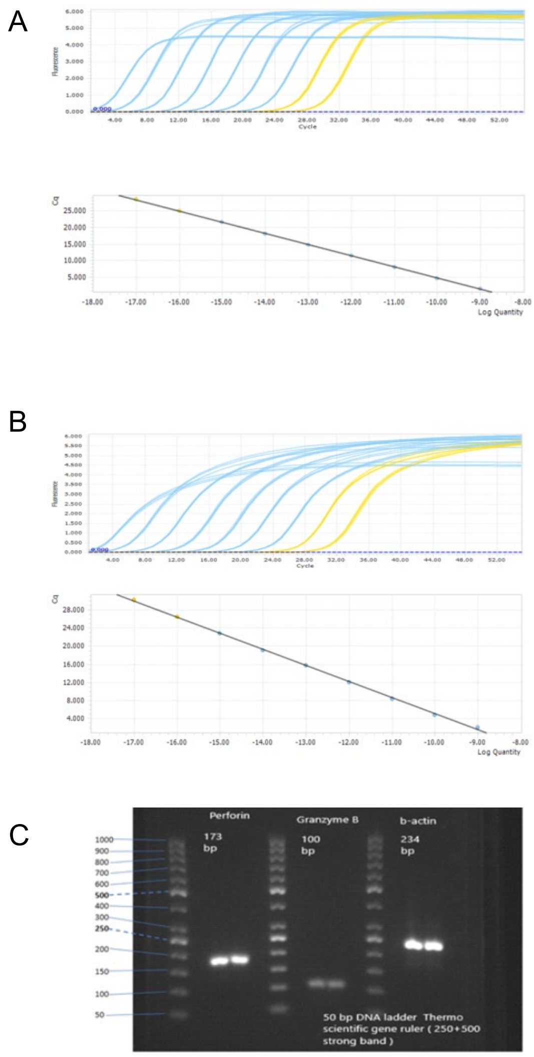

Suppl. Figure S1. Efficiency curves for Perforin (**A**) and Granzyme B (**B**) transcripts in mononuclear cells. For Perforin the efficiency was 1.98 (slop, -3.37; Y-intercept. -28.99) and for Granzyme B it was 1.91 (slope, -3.47; Y-intercept, -30.70). (**C**) Agarose gel (2%) electrophoresis of PCR products from Perforin, Granzyme B, and β-actin together with Gene Ruler 50 bp DNA ladder at lanes 1, 6, and 11. The ladder comprises thirteen DNA fragments, with two strong reference bands highlighted with dotted blue lines (250 bp and 500 bp).

Suppl. Table S1. Primers used for qRT-PCR.

GENE NAME Number	Primer sequence (5′- 3′)	Product size bp
GRANZYME B	Forward: 5′ TGGGGGACCCAGAGATTA 3′	
	Reverse: 5′ TTTCGTCCATAGGAGACAATG 3′	100
PERFORIN	Forward: 5′ CAGAAGACCCACCAGGACCAG 3′	
	Reverse: 5′ GAGATAAGCCTGAGGTAGGCG 3′	173
ACTB (β-ACTIN)	Forward: 5′ GGACTTCGAGCAAGAGATGG 3′	
	Reverse: 5′ AGCACTGTGTTGGCGTACAG 3′	234

Suppl. Table S2. Protocol for serial dilutions of dsDNA for determination of primer efficiency.

TUBE	H2O	dsDNA	Conc.
1	90µL	10µL of 10-8	10-9
2	90µL	10µL of 10-9	10-10
3	90µL	10µL of 10-10	10-11
4	90µL	10µL of 10-11	10-12
5	90µL	10µL of 10-12	10-13
6	90µL	10µL of 10-13	10-14
7	90µL	10µL of 10-14	10-15
8	90µL	10µL of 10-15	10-16
9	90µL	10µL of 10-16	10-17

STOCK SOLUTION 100µL cDNA
Perforin: 212 ng/µL
Granzyme B: 79 ng/µL
Perforin: 1/0.212 = 4.7 µL
Granzyme B: 1/0.079 = 12.7 µL
100µL of 1µg dsDNA.
